# Perception and Experience of Sexual and Gender Minority Korean Youth in School Counseling

**DOI:** 10.1007/s10447-022-09490-0

**Published:** 2022-10-20

**Authors:** Hyun Ji Yi, Yun-Jeong Shin, Yeseul Min, Jinwon Jeong, Jilli Jung, Yoojin Kang

**Affiliations:** 1 Seoul National University Hospital, Seoul, Republic of Korea; 2 Department of Education, Seoul National University, 1 Kwanak-ro Kwanak-gu, Seoul, Republic of Korea; 3 Pennsylvania State University, University Park, State College, PA USA

**Keywords:** School counseling experience, Sexual and gender minority youth, South Korean students, Consensual qualitative research

## Abstract

To comprehensively explore the school counseling experience of sexual and gender minority (SGM) youths in South Korea, we interviewed 14 SGM youths about their school counseling experience and the climate of their schools toward SGM. Results showed that a hostile school climate and the accessibility, confidentiality, trustworthiness, and LGBTQ competency of school counselors drove the reluctance of these youths to receive school counseling services. Implications for the role of school counseling services for SGM youths were discussed based on the findings of this work and the suggestions offered by the interviewed SGM youths.

**Supplementary Information:**

The online version contains supplementary material available at 10.1007/s10447-022-09490-0.

## Introduction

Sexual and gender minority (SGM) youths continue to be exposed to harassment and assault in schools (Kosciw et al., 2020; McCabe et al., 2013). Discrimination and stigma can negatively influence the mental health of these youths and subsequently lead to high emotional distress (Almeida et al., 2009; Wilson & Cariola, 2020). School counseling is one of the most accessible solutions that help SGM youths buffer their psychological stress and receive support (Russell & Fish, 2016). However, within the unique context of South Korea, only around 11% of SGM students reach out to their school counselors regarding their sexual and gender identity concerns, and many report that the counseling they receive is not helpful (National Human Rights Commission of Korea, 2014). Therefore, the focus of this study is to investigate the school counseling experiences of SGM youths in South Korea and to understand their hesitation or dissatisfaction with their school counseling services using qualitative inquiry.

### School Counseling for Sexual and Gender Minority Youths

School counseling can play a crucial role in enhancing the mental health of SGM youths and changing their hostile school climate (Goodrich et al., 2013). School counseling should adopt social justice advocacy and a multicultural approach to secure the mental wellness of underprivileged populations, such as racial minorities and SGM (Kiselica & Robinson, 2001; Toporek & Daniels, 2018). Many researchers emphasized the importance of school counseling in supporting and protecting SGM youths at school and argued that school counselors, as gatekeepers, have a wide range of responsibilities ranging from educating both students and school staff to enforcing policies that promote a queer-inclusive school climate (Asplund & Ordway, 2018; Beck, 2016; Goodrich & Luke, 2009).

Beck (2020) used qualitative inquiry to explore the experiences of school counselors with LGBT students and found how they promote a queer-inclusive school environment. Specifically, Beck (2020) indicated that school counselors should know the culture of schools toward their LGBT students and lend a voice to this population. Strear (2017) used a Delphi method to investigate the recommendations of 14 school counselors and educators about how to make schools queer-friendly.

Though the above studies explored how school programs can create a supportive climate for SGM youths and provide practical strategies for empowering these youths at school, these studies are mainly based on the experiences and theoretical arguments of school counselors and not on the experiences of SGM youths themselves. Few studies provide data or information about the school counseling experiences of SGM youths. Exploring such experiences may help school counselors improve their skills in providing client-oriented services. Given that SGM youths’ perspectives on school counseling may differ from those of school counselors, the former should be explored independently (Goodrich & Luke, 2009). Therefore, this research aims to give voice to the marginalized SGM youth in the literature. Moreover, given the hostile environment for queer youths in South Korea, this study provides insight into their experiences within the context of the South Korean school counseling system.

### Sexual and Gender Minority Youths in South Korea

Compared with western countries that have shown increased public support for the SGM population (Baunach, 2012; Witeck, 2014), the attitudes and perceptions toward SGM remain conservative in South Korea (Rich et al., 2021). Although the neighboring country of Taiwan became the first Asian country to legalize same-sex marriages in 2019, South Korea is facing strong resistance to these unions (Krumbein, 2021; Rich, 2017). Since 2007, the opposition has blocked the country’s attempts to pass an anti-discrimination law that protects SGM (Rich, 2017). In addition, conservative Christian communities in South Korea continuously reject homosexuality and HIV issues (Cho & Sohn, 2018; Jung & Phillips, 2017), and the dominance of Confucianism in the country, which emphasizes the importance of blood lineage and family honor, has shaped conservative attitudes toward SGM (Jung, 2020). These factors may explain why the attitudes and perceptions toward SGM in South Korea remain conservative compared with those in other countries.

In this social context, SGM youths in South Korea face greater marginalization than their adult counterparts. SGM youths have been portrayed as people living invisible without being recognized for their existence for quite some time (Kang & Kim, 2006). For instance, 92% of SGM youths in South Korea hide their true sexual orientations or gender identities out of fear of being harassed or assaulted by their peers (National Human Rights Commission of Korea, 2014). Some of these youths drop out of school and even attempt suicide due to continued harassment (Kang & Kim, 2006; National Human Rights Commission of Korea, 2014). Despite the rampant discrimination against SGM youths at school in South Korea, the country has few legal policies and school regulations that protect this population. In addition, the Ministry of Education in South Korea excludes sexual orientation- and gender identity-related topics from the country’s secondary sex education curricula (Um, 2018). This series of phenomena suggests how Korean society has remained silent and paid little attention to the population.

### School Counseling System in South Korea

School counseling systems in South Korea have several unique characteristics. Some school counselors in South Korea cannot actively intervene in school management due to the various employment conditions and statuses for each kind of school counselor (Lee & Oh, 2016). School counseling services in South Korea are usually provided by (1) professional counseling teachers, (2) career guidance teachers, (3) classroom teachers, and (4) professional counseling specialists (Choi & Lee, 2018). Unlike professional counseling teachers, career guidance teachers, and classrooms teachers who possess teaching licenses and hold teaching positions, professional counseling specialists who possess only counseling licenses and undergo only counselor training are not regarded as teachers and are only employed part-time in schools to provide counseling services to students; therefore, this system limits professional counseling specialists from actively intervening in school management (Lee & Oh, 2016).

A shortage of school counselors in South Korea is evident. Schools with more than two counselors in South Korea are rare (Sung & Lee, 2017). Only 13.3%, 46%, and 47.2% of elementary, middle, and high schools in South Korea have professional counseling teachers or counseling specialists, respectively (Korea Educational Development Institute, 2020). This trend does not follow the recommendations of ASCA, which indicates schools to have one counselor per 250 students (ASCA, 2012). The lack of school counselors may lead to difficulties in managing each student’s concerns.

A stereotype that school counseling services are for at-risk students in South Korea is a common stigma. Schools in South Korea have counseling hubs called WEE classes or WEE centers that provide counseling services to at-risk youths (Lee & Trevisan, 2019). Therefore, students may stay away from school counseling services.

### Research Questions

The purpose of this study is to understand the school counseling experiences of South Korean SGM youths. Our research questions are as follows:

- Why do SGM youths hesitate to receive school counseling services?
- What do SGM youths experience when receiving school counseling services?
- What do SGM youths want from school counseling services and their school counselors?

SGM youths in South Korea may experience difficulties coming out to parents due to the hostile climate toward the SGM population. Therefore, we decided to interview SGM youths aged 18 to 23 years who can determine research participation on their own without parents to protect participants from being outed, even though we may obtain retrospective memories. Considering that participants might lose the memories of school counseling experiences over time, we limited the age of participants to 23.

## Methods

Given the limited number of studies that explore the school counseling experiences of SGM youths, the researchers adopted the consensual qualitative research (CQR) approach, which is ideal for studying the inner experiences, attitudes, and beliefs of individuals, all of which cannot be readily detected by using traditional quantitative methods (Hill, 2012). By interviewing participants, researchers can obtain in-depth information that is not readily observable. CQR also allows researchers to embrace the uniqueness of their participants’ experiences and capture similarities in such experiences (Hill, 2012). Especially, CQR has been widely used to explore underrepresented minority individuals’ experiences, such as attitudes toward HIV-positive status disclosure among SGM individuals (Sauermilch et al., 2022), experience and coping during the school-age years with transgender people of color (Simons et al., 2021), racialized experience in Christian higher education among Asian American faculties (Hyun et al., 2022), and discriminated experience with Muslim Arab American women (Alsaidi et al., 2021).

### Participants

A total of 14 self-identified SGM youths aged 18 to 23 years were recruited as participants (Table 1). Among 14 participants, three had been assigned female sex at birth and identified as non-binary or gender non-conforming, three identified as transgender men (had been assigned female sex at birth and identified as men), and one identified as a transgender woman (had been assigned male sex at birth and identified as female). Regarding sexual orientation, three identified as asexual, two identified as lesbian, two identified as pansexual, one identified as bisexual, one identified as demisexual, one identified as questioning, and four declined to identify with a specific category. Regarding the experience of receiving school counseling, 70% of (n = 10, 71%) reported experiencing it at least once. Among participants who received school counseling, the majority of them reported disclosing SGM-related issues to school counselors. All these participants spent their schooling days in different locations across South Korea, and their schools had at least one counselor. Among these participants, 11 participants had received counseling from a school counselor.

**Table 1:** Participant Demographics

No	Age	Sexual or Gender Identity	Length/Frequency of receiving school counseling experience	Disclose SGM-related issues to school counselors
1	21	FTM Transgender, questioning	None	No
2	20	Lesbian	2 years	Yes
3	22	Genderqueer, Non-binary, Panromantic, Demisexual	7 years	Yes (once)
4	23	Panromantic Asexual	Once	Yes
5	21	Bisexual	None	No
6	22	Panromantic, Asexual	12 years	Yes (once)
7	23	FTM Transgender	Once	Yes (once)
8	20	Lesbian	None	No
9	22	MTF Transgender, Pansexual	Once	No
10	21	Biromantic Asexual	Once	No
11	18	Declined to identify with a specific category	None	No
12	18	Nonbinary, Panromantic, Pansexual	3 years	Yes (once)
13	19	FTM	3 years	Yes
14	18	Nonbinary	6 years	Yes

### Researchers

The research team included three doctoral students, one master’s student, one bachelor’s degree holder, and one professor in counseling who served as an auditor (for six cisgender women, of which four were heterosexuals, one was bisexual, and one was unsure) with extensive experiences in CQR and qualitative research. A key component of CQR is bias reporting and analysis (see Hill et al., 2005). Before the data collection, we reviewed the related CQR and research on the experiences of SGM youths at schools with counseling services and then shared the motivations for the study and the biases that may be associated with our respective backgrounds.

### Procedure

Institutional Review Board approval was obtained at the authors’ affiliation. We distributed recruitment advertisements to 14 SGM college student organizations and SGM rights organizations, posted these advertisements on our social media, and used the snowball sampling technique through personal contacts. The potential participants were informed about the purpose and procedures of this study and were screened for their eligibility based on the following criteria: (1) had concerns about their gender identity and/or sexual orientation during middle and/or high school; (2) attended middle and/or high schools with counselors; (3) a Korean citizen aged 18 to 24 years; (4) and voluntarily participated in the study.

All participants signed a consent form before the interviews. Given that this study was conducted during the COVID-19 pandemic, the interviews were conducted in person or via ZOOM, depending on the participants’ preferences. By following Hill’s recommendation, we tried to use eight to 10 open-ended scripted questions per hour, so each interview lasted between 60 and 90 min and followed a semi-structured protocol with six main scripted questions, where the participants were asked about their school counseling experiences and the climate of their schools toward SGM youths (see Appendix). This interview protocol was created after reviewing the previous literature and sharing our thoughts and research experiences. Specifically, we tried to develop interview protocol questions to explore SGM students’ perceptions about school counseling and their reasons to receive or not receive school counseling by discussing possible probes prior to interviews and practicing probing by conducting pilot interviews. Each interview was recorded, and the recordings were transcribed before they were discarded.

After transcribing the interviews, each researcher independently read the transcripts to develop domains (i.e., topic areas) and then consensually made the first domain/category (i.e., themes) list. The interview transcripts were then classified according to these domains and categories, and the discussions continued until the list was deemed stable. During the cross-analyses, each researcher reanalyzed different domains and revised the domain, category, and subcategory lists until a consensus was reached. In all discussions, the domains and themes were reviewed and audited by the professor researcher. All data were coded into CQR terminology: general (all or all but one case; 13–14 cases), typical (half or more than half; 7–12 cases), and variant (at least three participants up to less than half; 3–6 cases).

Prior to the interview and data analysis, researchers implemented several steps to ensure the trustworthiness of the qualitative study. We tried to follow the strategies, such as sharing bias, recording reflexive journaling, discussing with research team members regularly, and utilizing an external auditor to secure the trustworthiness, as recommended by Lincoln & Guba (1986) and Hays & Singh (2012). First, researchers shared their own biases regarding the SGM population, heterosexism, and cisgenderism and expectations about this study for three months on a biweekly basis before the interview. For instance, we reminded ourselves to be cautious about viewing SGM youths as vulnerable individuals that must always need counseling services. Second, researchers reviewed and presented journals related to SGM youths in South Korea, LGBTQ + counseling, and the school counseling system in South Korea to understand the current situation of school counseling for SGM youths in South Korea. Third, five researchers participated in the whole data analysis process for cross-checking the results. One professor in counseling who has numerous research experiences related to multicultural counseling, including SGM issues in South Korea, audited the data analysis.

## Results

We analyzed 14 interview data to understand South Korean SGM youths’ perceptions toward and experiences with school counseling services. We identified four domains and 23 categories from our data (Table 2).

**Table 2:** SGM Youth’s School Counseling Experience

Domains/Subcategories	Frequency
**Living as an SGM adolescent** Heterosexual school climate School curriculum that bypasses SGM Teacher’s discriminatory/lukewarm attitudes toward minority Teacher’s openly supportive attitude to minorities Peer’s disgusting remarks toward SGM Peer’s careful/caring attitude toward SGM Feel comfort by recognizing the existence of another SGM peer Family’s unaccepting attitude toward SGM Confusion in the face of heteronormative world-denying one’s identity Adaptive cognitive-behavioral strategies to self-protection	14/General 11/Typical 14/General 7/Typical 13/General 6/Variant 6 /Variant 7/Typical 11/Typical 13/General
**Context of Counseling utilization** Negative perception of school counseling Various reasons for visiting the school counselor	9/Typical 12/Typical
**Experiences in the Counseling process** Wondered whether or not to bring up minority issues Minimizing reactions from the school counselor Accepting reactions from the school counselor Functioned as a solace, but SGM issues were overlooked Acknowledged limitations of school counseling Sought out-of-school counseling services	10/Typical 7/Typical 2/Rare 9/Typical 4/Variant 8/Typical
**Suggestions for school counseling** Create an SGM friendly atmosphere Lower barriers for counseling Teacher’s affirmative attitudes toward SGM School counselor’s role as an SGM advocate Sexual minority rights education for students	8/Typical 4/Variant 7/Typical 10/Typical 7/Typical

### Domain 1: Living as an SGM Adolescent

#### Heterosexual School Climate

SGM youths generally feel that their school atmosphere does not welcome multiculturalism and diversity. The participants shared that topics related to minorities, such as sexual orientation and gender identity, were not even mentioned in their schools as if they did not exist at all. Participant 1 faced difficulty sharing her sexual identity with her friends because “Teachers or adults do not bring up these things [minority issues] first, so students just keep their mouths shut and treat the minorities as non-existent.” Participant 5 felt a “heterosexuality-oriented atmosphere” among his peers and shared, “I felt like I had to talk about men as if I was interested in men, so I pretend that I like male idol groups.”

#### School Curriculum that Bypasses SGM

The participants recalled that their school curricula did not recognize the existence of sexual minorities. For example, Participant 10 commented, “I have never seen anyone who was taught about that topic throughout elementary, middle, and high school.” Even their topics in sex education classes were limited to pregnancy and childbirth and never mentioned anything about sexual minorities. According to Participant 7, “They were only telling us things like ‘Don’t have an abortion’ or ‘Put on a condom like this.’ These classes only dealt with sexual relationships between men and women engaged in ‘Heteronormativity.’”.

#### Discriminatory/Lukewarm Attitudes of Teachers Toward the Minorities

The participants were generally frustrated about the discriminatory remarks of their teachers against gender minorities. For example, Participant 7 remembered his teacher’s words as follows: “He said that homosexuality has a bad influence on students and society. He also said that health insurance premiums had become expensive due to the AIDS epidemic and homosexuality.” Meanwhile, the other participants described their teacher’s lukewarm feelings of disgust. For instance, Participant 6 never witnessed “any teacher who scold students for making hate speeches.“ Participant 2 recalled an episode with a teacher who restrained her advocacy for groundless reasons, saying, “I uploaded a book from the Gender Minority Human Rights Alliance in my cumulative student records, but they told me, ‘If you upload this, you may be at a disadvantage when you apply for university admission.’”.

#### Openly Supportive Attitude of Teachers to Minorities

In contrast to the aforementioned experiences, the participants typically relied on teachers with open-minded attitudes toward SGM. Some of their teachers took active actions, such as teaching their classes about minority rights or connecting their students to counseling resources. Participant 14 shared, “He recommended the ‘DDING DONG’ (the only LGBTQ youth crisis support center in South Korea) counseling service… He reminded us that being queer is not always a heavy and taboo topic when he taught us about minority rights in class.” Participant 11 also had a reliable teacher who “…encouraged me to join club activities for SGM. When I saw queer books in her office, I was assured that she was a person who believes in the equal rights of all people.”

#### Disgusted Remarks of Peers Toward SGM

The participants directly faced hate speech and microaggression from their peers. For instance, Participant 7 shared that during his school days, “If a girl wears pants to school instead of skirts or cuts her hair too short, others would immediately start a rumor that she is a ‘lesbian.’” Few participants also heard their classmates expressing their disgust toward SGM. For instance, Participant 1 shared, “Some derogatory words toward SGM, such as ‘you look gay’ or ‘are you gay,’ were often used by boys to tease and ridicule one another, but no one ever stopped them from using those words.”

#### Careful/Caring Attitude of Peers Toward SGM

In contrast to 13 participants who felt excluded for being SGM, 6 participants received support from their peers. For instance, Participant 2 expected that her friends would bully her, but surprisingly, “They did not support me openly, but I felt like they were trying not to hurt me by choosing their words carefully. So I was uncomfortable and grateful at the same time.” Meanwhile, Participant 6 shared, “Some of my friends hated listening to hate speech and complained about it.”

#### Feel Comfort by Recognizing the Existence of Another SGM Peer

Some participants found a supportive network in queer communities where they could communicate freely about their identity. One participant described this community as a “dream place” whose members always think positively about SGM. By having friends who share the same thoughts, the participants were able to recover from the emotional harm they experienced from others’ exclusive attitudes (Participant 9) and enjoy queer culture (e.g., queer parades) together (Participant 11). The participants also took comfort in the idea that other SGM people are in their surroundings. Acknowledging the presence of SGM peers in their daily lives helped these participants confirm their sense of universality and belonging. According to Participant 12, “I was so lucky. I didn’t know at first, but it turned out that all my close friends were queers.”

#### Unaccepting Attitude of Family Toward SGM

Exclusivity toward SGM was not confined to schools. Many adolescents reported that their identities were not supported or affirmed even at home. Even after disclosing their identities, many participants reported that their parents continued to deny or correct their identities. For instance, Participant 12 mentioned that their mother, who was educated on minority rights while working as a teacher, once exclaimed, “Well, I suppose that being pansexual is better [than being homosexual].” Meanwhile, Participant 14 shared how her parents refused to acknowledge her sexual identity: “One day, my mom suddenly checked my cellphone. She looked through my photos and asked me, ‘Who is she? Why did you take pictures of you hugging each other?’ I made an excuse by saying, ‘She is just a friend,’ but my mom became upset and started hurling hate speeches at me, like, ‘Why are you dating a girl? Are you out of your mind?‘”.

#### Confusion in the Face of a Heteronormative World Denying One’s Identity

During their process of identification, the participants typically experienced internal confusion due to their sexual identity. They often thought, “I’m weird, and other people are normal” (Participant 6). They were constantly worried about being rejected in a relationship. Participant 1 reported that she wanted to talk to her SGM friends about a common topic to feel a sense of belonging. She used to brood on the question, “Is there anyone like me in this school? I want to find such a friend.”

#### Adaptive Cognitive-Behavioral Strategies to Self-Protection

The participants were not merely passive and actively protected themselves from external discrimination in various ways, such as deciding not to disclose their sexual identity, finding a supportive community, speaking up for advocacy, and correcting misunderstandings about SGM. For instance, Participant 14 shared, “I told the counselor that she could use the word ‘partner’ rather than ‘boyfriend’ and ‘girlfriend.’” These participants all used acting to promote the rights of SGM people. According to Participant 14, “There are many SGM people in our daily life, but their beings are always buried. If they can come to the surface even by a little because of me, I will always participate in studies like this.”

### Domain 2: Context of Counseling Utilization

#### Negative Perception Toward School Counseling

Most of the participants described school counseling as a resource that their peers would not willingly access due to low trust toward its confidentiality and “the stigma that students with a bad reputation go there” (Participant 12). Participant 8 refused her school’s counseling service because “I’m afraid that everything I say about coming out or having a hard time mentally will be shared with my parents or even teachers.” Meanwhile, Participant 2 shared, “I went to a WEE class in secret. The class seemed like a place where troubled students go as a last resort. I felt branded just by going to that class.”

#### Various Reasons for Visiting the School Counselor

The participants typically visited their school counseling center seeking for help on problems other than SGM issues (e.g., academic concerns, relationship with parents, and friendships). The reasons behind their visits were also diverse, such as voluntary visits, recommendations from teachers, or forced visits. For Participant 3, “I went to the counseling service mainly because I had concerns about domestic violence.” For Participant 7, “There was a mandatory psychological test for all students in the school. Because I had a high level of depression, I was forced to take counseling.” However, Participant 2 stressed that sexual identity issues are inevitably linked to other difficulties related to school adjustment. She shared, “Sexual orientation acts as a starting point that affects family relationships and promotes a sense of alienation. I was always worried that my friends might find out about my sexual orientation or write a story about me behind my back on social media. That’s all connected. When I affirmed my identity, I thought that everything would go wrong from there.”

### Domain 3: Experiences in the Counseling Process

#### Wondering Whether or Not to Bring Up Minority Issues

In the counseling process, the participants went through different phases when deciding whether to come out to their counselors or not. Some refused to bring up topics related to minority identity in fear that their counselors would catch signals that they are faithful Christians (e.g., wearing cross necklaces), whereas others were unsure that anything they shared with their counselors would be kept confidential. Participant 3 was afraid of ruining their relationship with their counselor. “I liked my counselor so much, and I didn’t want to hear her saying that I’m the weird one. I wanted to keep our nice counseling sessions going, and I didn’t tell her about my identity because I knew I’d be disappointed.” By contrast, some participants’ disclosure came naturally because they could not leave out their sexual identity when talking about themselves. Some of them had full trust in their counselor, such as Participant 2, who said that “It seemed like she could listen to everything.”

#### Minimizing Reactions from the School Counselor

Seven participants who disclosed sexual identity concerns during their counseling reported that the counselors were surprised, did not take them seriously, and even conveyed disgust. These participants were dumbfounded, disappointed, offended, and resolved not to get their hopes up again. One example negative response was, “You don’t have to do that. That’s not normal” (Participant 3). Meanwhile, other counselors shared that these participants would eventually grow out of such a childish phase by saying, “It’s because you cannot see many boys around you, and that’s why girls get interested in each other… It seems like friendship to me” (Participant 4).

#### Accepting Reactions from the School Counselor

Few participants shared that their counselors were not surprised about their identities and even responded with an accepting tone. Participant 13 felt that their counselor “took it in” after explaining about gender dysphoria. Despite describing the phenomenon as a cliché, Participant 12’s counselor argued, “People are all equal. It’s not a bad thing.”

#### Functioned as a Solace, but SGM Issues Were Overlooked

Counseling functioned as a shelter where participants could turn for emotional support, but most of the participants felt stuffed after their counseling sessions because “at the end of the day, I couldn’t tell the whole story” (Participant 5). They could talk to their counselors about anything except for their sexual identity concerns. Although school counseling was used by students to avoid their classes or to drop by for sweets during their lunch or breaks, they also described their school counseling offices as a place where they could “hide in the midst of that hell” (Participant 13). The participants agreed that counseling did not fundamentally help them in their struggles. According to Participant 2, “I thought my fundamental problems were my sexual orientation and romantic relationship, and the family conflicts that followed. But the counselor overlooked all that and just oversimplified my problem as wanting to quit school.”

#### Acknowledged Limitations of School Counseling

Some participants addressed the structural limitations of school counseling. For instance, some school counselors only held part-time positions and were only available on certain days. Therefore, the participants complained that scheduling an appointment was “difficult because the number of students going to the WEE center is much larger than you might think” (Participant 2). Meanwhile, Participant 9 shared that counselors do not have much influence on the school climate by saying, “The WEE class counselor is called a teacher, but in the school, she is not accepted as one of our subject teachers. She’s not here all day, and she has other work outside the school too. There isn’t much she can do about the things that are going on at school.” Limitations in confidentiality were also a concern. Participant 6 shared, “I heard that the things we say in counseling are being passed to the other teachers… I realized that my counselor was not the only one listening. They were taking records, so I couldn’t really trust their confidentiality.”

#### Sought Out-of-School Counseling Services

The participants typically sought other counseling services, such as psychiatry clinics, queer-friendly private counseling clinics, and other youth counseling services provided by the government. They were sometimes referred to these services by their school counselor or were dragged by their close friends (Participant 9). They felt that out-of-school counseling services were more confidential than those offered at their schools, and they were relieved that they could “come out straight away” (Participant 10). Counselors in queer-friendly institutions were willing to offer them support in exploring their identities. Participant 12 admitted, “While the school counselors are like ‘okay, homosexuality is different, but I can accept it,’ the counselors outside the school seemed to actually know about these concepts.”

### Domain 4: Suggestions for School Counseling

#### Create an SGM-Friendly Atmosphere

The participants highlighted the need for an SGM-friendly school climate by stating, “Acknowledging our existence would be the place to start” (Participant 4). Participant 1, who did not receive school counseling, claimed that “Even if I did know that there is a counseling center at my school, I couldn’t just barge in there and say ‘This is who I am’ when I am not even sure that the counselor is an ally or not. I think it’s really important for the school counselor and the entire school atmosphere to be open and clear: Our school is queer-friendly.” The participants suggested posting explicit signs, such as stickers, rainbow flags, and pride month notices, throughout the campus and installing inclusive school facilities, such as unisex toilets. They also proposed creating an on/offline community or network “where queer adolescents can meet and be who they are” (Participant 6).

#### Lower Barriers for Counseling

Four participants called for a change in the negative perceptions toward people who receive counseling so that SGM individuals will “not have to worry about how other people would see them” (Participant 12). Counseling centers should be a place where anyone can go regardless of their concerns. When promoting their counseling services, schools should “tell their students that they can receive counseling for gender identity and sexual orientation issues and talk about their parents, study, career, and friendships. Just one or two extra phrases may help the youth realize that they can go and confide themselves in their school counselors” (Participant 14).

#### Affirmative Actions of Teachers Toward SGM

The participants typically referred to teacher support and affirmative actions and emphasized their strong influence on students. According to Participant 6, “I wish that teachers would not be so obvious even if they do think badly about it. The school space is all the students have. There’s nowhere they can run to when they are refused access to this space, and such denial may make them give up their expectations altogether. I hope that teachers would listen to the concerns of their students.”

#### Role of School Counselors as SGM Advocates

The professional competence of school counselors was intertwined with their knowledge of sexual identity and willingness to advocate. For Participant 6, “The most important thing is to tell them that it’s not wrong, that using such kind of language is hate speech, and that you don’t need to be hurt by that.” Counselors need to give an impression of queer friendliness or surround themselves with pamphlets/flags in order to give students some feeling of reassurance. These counselors should also be resourceful enough to provide students with a list of other counseling institutions where they can obtain friendly support.

#### Sexual Minority Rights Education for Students

The participants agreed that sex education should take an affirmative stance and should cover “diverse forms of sexuality” (Participant 1). They were aware of how the current human rights/sex education in their schools backfired. They maintained that sexual minority rights education should include not only concrete information but also lessons on how to treat SGM youth with respect. Participant 9 suggested that instead of lectures, sexual minority rights education should be delivered through interactive discussions. They stressed that more students need opportunities to “discuss how to think about and how to relate to someone whose identity differs from theirs.” The participants contended that simply acknowledging the presence of diverse sexualities would empower SGM youths and support them in discovering their identities.

## Discussion

We identified individual and environmental factors that influence one’s decision to receive or refuse school counseling services and the counseling process itself. One of our noteworthy findings is that a hetero-cis-normative school climate toward SGM-related content is a distal factor that may affect SGM youths’ decision to receive school counseling services. Although some teachers and peers show supportive attitudes toward SGM youths, the majority of research participants reported that exclusive attitudes are dominating in schools, and they were sometimes excluded from their school curricula. Such a hostile school climate may have intensified the internal confusion of participants as they felt to have no choice but to face a hetero-cis-normative world that rejects their identity. Combined with such a discriminatory school climate with not knowing whether the counselor is an ally or not, participants experienced internal confusion, such as questioning their normality due to their identity, and this confusion seemed to have affected their hesitation to receive school counseling services. Given that one’s self-disclosure of stigmatized identity is influenced by their social climate (Chaudoir & Fisher, 2010), hostile environments may block SGM youths from coming out to their school counselors.

The negative perception toward school counseling services is another factor that explains the hesitation of SGM in receiving school counseling services. These youths refuse to receive school counseling in the current study mainly due to their concerns about the confidentiality and prejudice of such service. Given that coming out to others is challenging for SGM youths (Denes & Afifi, 2014; Goldfried & Goldfried, 2001), maintaining confidentiality is crucial in school counseling services. However, South Korean school counselors cannot guarantee the confidentiality of their sessions because homeroom teachers are deeply involved in their students’ issues, hence pressuring counselors to share details of their sessions with these teachers (Lee & Yang, 2008). Given such a lack of confidentiality, participants in this study were afraid of disclosing their sexual identities to others and refused to receive school counseling. They also hesitated to receive school counseling services because of the prejudice associated with school counseling services. Given that school counseling in South Korea applies a remedial approach than a preventive one (Lau & Fung, 2008), a problem-based model may intensify the prejudice that students who receive school counseling services are troublemakers. These negative perceptions of school counseling may influence SGM youths’ hesitation to receive school counseling.

In the process of school counseling, SGM youths may consistently test if they can show their true selves to their school counselors. The participants refused to bring up sexual-identity-related issues to their counselors mainly out of fear of receiving negative reactions. Some school counselors give discriminatory remarks to SGM youths in this study, who in turn, cannot formulate a proper response to such remarks. Therefore, school counselors should actively advocate the rights of SGM youths to give them some reassurance. This suggestion is aligned with the arguments of previous studies that emphasize the social justice advocacy of counselors for their clients, which represents a fifth force of counseling (Ratts et al., 2010). However, some participants discussed sexual-identity-related issues with their counselors despite worrying about their negative reactions because they believed that such issues were not taboo and the other concerns being discussed were somehow related to these issues. Moreover, not all counselors reacted offensively to these issues; one of them even held an accepting attitude.

Another interesting finding is that SGM youths may not perceive school counseling as a fundamental solution. The participants were gratified with the empathy and support of their school counselors to some degree, hence perceiving these counselors as a safe place where they can address some personal concerns. However, these counselors tend to overlook SGM-related issues. Therefore, these counselors should improve their counseling competence by understanding the SGM-related issues of SGM youths.

Some SGM youths also seek out-of-school counseling services in addition to school counseling. In other words, they seek help from mental health professionals and do not shun professional help altogether. This finding is consistent with those of Vogel & Wei (2005), who found that a lack of social support leads people to seek additional professional help to manage their psychological stress. SGM youths may have felt some limitations in school counseling, hence driving them to seek out-of-school counseling. School counselors also need to learn about the difference between their own counseling and out-of-school counseling to understand why SGM youths seek the latter.

The participants offered two suggestions for improving school counseling. First, many participants highlighted the importance of enhancing the professional competence of school counselors to improve the quality of school counseling services for SGM youths because some of these counselors lack basic knowledge about SGM and even give discriminatory remarks. Compared with other countries such as the US, South Korea lacks training models and programs that school counselors can use to enhance their SGM counseling competence (Beck et al., 2014). Therefore, the government or the Department of Education of South Korea should provide the necessary training courses for these counselors. Moreover, decorating school counseling centers with SGM advocacy objects (e.g., rainbow flags) can turn these places into safe spaces for SGM youths and subsequently encourage their self-disclosure (Chaikin et al., 1976). The negative perceptions toward people who receive school counseling services should also be addressed.

Second, the participants wished for changes in various aspects, such as the attitudes of teachers and peers toward SGM and the school curricula. To change the perceptions of teachers and peers toward SGM, SGM-related education (e.g., sex education) for students and teachers is highly demanded. Adding SGM-related content in sex education or other subjects may contribute to changing the negative perceptions of peers and teachers toward SGM youths. Furthermore, visual markings (e.g., rainbow stickers and flags) and networks (e.g., Gay–Straight Alliance) that support SGM youths at school can help alleviate the hostility of school environments toward these youths. Through these changes, SGM youths begin to perceive their schools as safe places, thus encouraging them to avail school counseling services.

### Implications for Practice and Research

This study offers some practical suggestions on how to promote school counseling services for SGM youths. First, school counselors should provide both direct and indirect services to enhance school counseling services for SGM youths. Previous studies have emphasized the role of school counselors in changing the school climate and in improving the quality of school counseling services (Abreu et al., 2016; Asplund & Ordway, 2018). School counselors in South Korea have been encouraged to take on the role of providing direct counseling services (Lee et al., 2007). Meanwhile, their role in providing indirect services, such as being advocates for SGM youths and changing the negative perceptions of teachers and students toward SGM youths, has been largely overlooked. The ASCA model suggests that school counselors should advocate for their students by cooperating with other teachers, parents, and school staff and contributing toward changing the school system (ASCA, 2012). Therefore, the role of South Korean school counselors should be expanded to promote school counseling services.

Furthermore, school counselors should positively promote school counseling services to reduce the barriers faced by those students who wish to avail of such services. SGM youths are concerned about being stigmatized as troublemakers due to the prejudice associated with receiving school counseling. For example, school counselors can promote the positive effects of receiving school counseling services and their openness in discussing various issues, including those related to SGM. The school counseling model should also be transformed into a preventive model (Lau & Fung, 2008) to encourage students to avail school counseling services without worrying about the associated stigma.

The participants were dissatisfied with those school counselors who lacked knowledge about SGM issues. Therefore, school counselors should learn basic knowledge about SGM youths and the related terms. As Asplund & Ordway (2018) urged school counselors to educate themselves, teachers, and students about LGBTQ issues, learning about topics of queer-related issues is crucial for school counselors themselves. ASCA (2016) also suggested school counselors to enhance the well-being of underprivileged students by providing them support and resources. Therefore, school counselors should improve their social justice counseling skills, especially for SGM youths, by forming a study group with other school counselors. They should also provide information about SGM-related issues because SGM youths want to know more about themselves.

However, sole effort from school counselors is insufficient. The effort of school counselors should be accompanied by political support from the government or state when they make SGM-friendly school programs or policies (Russell, 2012). The government should provide consistent training or supervision to enhance counseling competency, including social justice and advocacy counseling competency. Since there are several ways to be school counselors in South Korea, such as being a professional counseling teacher, career guidance teacher, or counseling specialist, the training models for school counselors are also different (Sung & Lee, 2017). For example, in terms of training, all school counselors in the US are required to complete a 700-hour practicum. However, the amount of required practicum is not unified among professional counseling teachers, career guidance teachers, and counseling specialists in South Korea (Yu, 2007). Most importantly, regardless of the difference in training period and time, there is a lack of training on increasing and enhancing social justice counseling competency in South Korea. Only three out of 17 metropolitan/provincial offices of education in South Korea specify SGM as one of the minorities who are guaranteed their rights under the Ordinance of Student Rights, and only one region, Seoul, has specified SEM in the Comprehensive Plan for Student Rights (Lee, 2021). In particular, the Seoul Metropolitan Office of Education(SOME) announced the plan to provide counseling support when SGM students are subjected to human rights violations such as discrimination and hatred last year. Thus, the training model for school counselors needs to be developed and operated to improve social justice and advocacy competency for promoting the mental health and human rights of SGM students in the future.

Furthermore, the government or state should make policies or anti-discrimination laws to protect SGM youths and include SGM-related issues in the regular curriculum. The government and state should educate the school principals and school staff because they make the final decision on school policies. School counselors can advocate for SGM youths in various ways through the heightened perception of the school principals and school staff toward SGM-related issues.

Governmental efforts to improve the quality of school counseling services are likewise needed. Unlike ASCA’s recommendation encouraging one school counselor to look after 250 students (ASCA, 2012), there is only one counselor per school, and even the rates of schools with professional school counselors assigned per school are still low in South Korea(Korea Educational Development Institute, 2020). Therefore, we recommend that the government increase the number of professional school counselors on a national scale and reduce the heavy workload, such as administrative work, so that school counselors can care for more underrepresented students.

Our findings can help expand research on the school counseling of SGM youths. Unlike previous studies that highlight the opinions of school counselors about the counseling of SGM youths, we convey the voices and opinions of SGM youths about this service. We mainly investigated the school counseling experiences of SGM youths in South Korea, where a domestic anti-discrimination law has not been enacted due to fierce opposition. Therefore, our study can guide other countries that hold conservative attitudes toward SGM issues.

### Limitations and Future Research

Although this study highlights the school counseling experiences of SGM youths in South Korea, some limitations must be noted. First, our sample may not represent the general SGM youths in South Korea because some of these youths may be hidden. Nevertheless, we tried to include a broad range of samples by selecting SGM youths with various identities. Future research should involve more participants to represent SGM youths.

Second, given that most of our participants reported that school counseling was unhelpful, we could not fully explore the advantages of receiving school counseling for SGM youths. The participants also did not receive school counseling services in the long term during their adolescence. Therefore, we could not thoroughly explore the positive effects of school counseling. Future research should explore the positive aspects of school counseling by interviewing SGM youths who regard school counseling experience as supportive and those who have been receiving school counseling services for an extended period.

Third, our study was based on retrospective experiences. Although we recruited participants aged between 18 and 23 years to minimize the period of recollection of adolescence, we partly depended on the participants’ memories during their adolescence. Therefore, future research should interview SGM youths during their adolescence.

In sum, the current study is meaningful in that it explores the reasons and experiences of using or not using school counseling by examining the situation of SGM advocacy counseling in South Korea from the perspective of LGBT youth. Our findings may serve as a cornerstone for fostering a positive and safe school environment for SGM and facilitating the follow-up research and discussion to strengthen the LGB advocacy counseling capacity of school counselors.

## Electronic Supplementary Material

Below is the link to the electronic supplementary material.

Supplementary Material 1

## Data Availability

The datasets used and/or analyzed in this study are available upon reasonable request from the corresponding author.
