# Supplementary material for: Perception and Experience of Sexual and Gender Minority Korean Youth in School Counseling
Source: Int J Adv Couns. 2022 Oct 20;45(2):189–209. doi: 10.1007/s10447-022-09490-0 (PMC9581756; doi:10.1007/s10447-022-09490-0)
Supplement: Supplementary file 1 — Supplementary Material 1 [file 10447_2022_9490_MOESM1_ESM.docx]

Appendix. *Interview protocol*

| **Ⅰ. Participants *not receiving* school counseling** |
| --- |
| 1. What was a perception of and attitude toward LGBTQ people within your school?  (specifically, school lessons/rules, attitudes of teachers and friends toward LGBTQ)  1.1. What was a teacher’s attitude when giving sex education or human rights education? |
| 2. What concerns would you want to address during school counseling?  (before this question, researchers need to check participant’s perception of school counseling and whether or not the participant wanted to receive school counseling)  2.1. What did you consider receiving school counseling? |
| 3. Did you want to address your concerns about sexual identity in school counseling?  3.1. If so, why did you not receive school counseling?  3.2. If not, why didn’t you want to address your concerns in school counseling? |
| 4. Have you ever received counseling on your sexual identity at other counseling centers?  If so, why did you consult with an outside counselor not belong to your school?  4.1. What was the difference between the school and other counseling centers? |
| 5. How did you relieve the problem stemming from your sexual identity? |
| 6. What should be changed to promote LGBTQ students’ use of school counseling?  6.1. Have you received counseling in college? If so, why?  6.2. What should be changed to make schools queer-friendly? |
| **Ⅱ. Participants *receiving* school counseling** |
| 1. What was a perception of and attitude toward LGBTQ people within your school?  1.1 What was a teacher’s attitude when giving sex education or human rights education? |
| 2. What concerns would you want to address during school counseling?  2.1. What do you think of school counseling?  2.2. What was your motivation to receive school counseling? |
| 3. What kinds of expectations/worries about sharing your concerns on sexual and gender identity during school counseling? |
| 4. Have you ever shared your concerns about sexual and gender identity during school  counseling?  4.1. If so, why?  4.2. What was the counselor’s attitude and/or reaction when you talked about issues relating to your sexual identity?  4.3. What help did you get from the school counseling? If not, why have you not shared? |
| 5. What should be changed to promote LGBTQ students’ use of school counseling?  5.1. Have you received counseling in college? If so, why? |
| 6. What has changed after receiving school counseling (positive, negative)  6.1. Would you recommend school counseling to LGBTQ students? Why or why not? |
